# **-Postprandial pancreatic [^11^C]methionine uptake after pancreaticoduodenectomy mirrors basal beta cell function and insulin release

**DOI:** 10.1007/s00259-016-3451-0

**Published:** 2016-07-07

**Authors:** Emanuel Steiner, Lukas Kazianka, Robert Breuer, Marcus Hacker, Wolfgang Wadsak, Markus Mitterhauser, Thomas Stimpfl, Birgit Reiter, Georgios Karanikas, Johannes Miholic

**Affiliations:** 1 Department of Surgery, Medical University of Vienna, Waehringer Guertel 18-20, A-1090 Vienna, Austria; 2 Department of Biomedical Imaging and Image-Guided Therapy, Division of Nuclear Medicine, Medical University of Vienna, Waehringer Guertel 18-20, Vienna, A-1090 Austria; 3 Clinical Institute of Laboratory Medicine, Forensic Toxicology, Medical University of Vienna, Waehringer Guertel 18-20, A-1090 Vienna, Austria; 4 Department of Biomedical Imaging and Image-Guided Therapy, Division of Nuclear Medicine, Divisional Head PET-PET/CT (Nuclear Medicine), Medical University of Vienna, Waehringer Guertel 18-20, A-1090 Vienna, Austria

**Keywords:** [^11^C]Methionine, Positron emission tomography, Pancreas, Gastric emptying, Insulin, Beta cell function

## Abstract

**Purpose:**

[*S-methyl*-^11^C]-L-methionine ([^11^C]MET) uptake in the pancreas might be a central indicator of beta cell function. Since gastric emptying was recently shown to influence glycemic control in subjects after pancreaticoduodenectomy (PD, the surgical treatment of neoplasms of the pancreas head), we looked for imaginable relationships between gastric emptying, pre- and postprandial insulin concentrations, and [^11^C]MET uptake.

**Methods:**

Nineteen tumor-free survivors after PD (age mean ± SD: 61 ± 8.7 yrs.; 10 male, 9 female) and 10 healthy controls (age: 27 ± 8.7 yrs.; 7 male, 3 female) were given a mixed test meal. One gram of paracetamol was ingested with the meal to evaluate the speed of gastric emptying. Insulin, glucose, and paracetamol plasma concentrations were measured before and over 180 minutes after ingestion. Beta cell function was calculated from fasting glucose and insulin plasma concentrations. Simultaneously, 800 MBq of [^11^C]MET were administered and the activity (maximum tissue standardized uptake values [SUVmax]) over the pancreas was measured at 15, 30, and 60 minutes after injection. Total integrated SUVmax (area under the curve [AUC]) and incremental SUVmax were calculated.

**Results:**

The uptake of [^11^C]MET in the pancreas was significantly higher (*p* < 0.0001) in controls compared to the PD group. Gastric emptying was significantly slower in controls compared to pancreatectomy subjects (*p* < 0.0001). Paracetamol AUC_30_ correlated with the SUVmax increment between 15 and 30 minutes (R^2^ = 0.27, p = 0.0263), suggesting a relationship between gastric emptying and the uptake of [^11^C]MET. Total integrated SUVmax correlated with insulin AUC_60_ (R^2^ = 0.66,*p *< 0.0001) in patients after PD. Multivariate regression analysis revealed insulin AUC_60_ and beta cell function, calculated from the fasting insulin to glucose ratio, as independent predictors of ^11^C-methionine uptake, i.e. total integrated SUVmax, in patients after PD (R^2^ = 0.78,* p *< 0.0001).

**Conclusion:**

Postprandial [^11^C]MET uptake may represent basal and postprandial beta cell function. The findings suggest a possible usefulness of this imaging procedure for further studying beta cell function.

## Introduction

Positron emission tomography (PET) is widely used to measure amino acid metabolism and protein synthesis rates in vivo. [*S-methyl*-^11^C]-L-methionine ([^11^C]MET), a PET tracer, radiolabeled with short-lived carbon-11 without any alteration to the molecular structure, is an universal donor of transmethyl reactions that participates in the synthesis of DNA and RNA. Methionine is mainly incorporated in the liver, brain, pancreas, and in tumors, where its main metabolic pathway is protein incorporation [1, 2]. Comar et al. suggested the use of [^11^C]MET PET imaging for the examination of the pancreas in 1976 [3]. Since then, various studies have addressed the relationship between tracer uptake, beta cell function, and exocrine pancreatic function [2, 4–11]. Syrota et al. studied [^11^C]MET uptake and protein incorporation in duodenal aspirate in patients with chronic pancreatitis, and observed a diminished uptake and incorporation of the tracer. Tracer uptake and amylase output after secretin stimulation were closely correlated [8, 9]. Similarly, Takasu et al. found a close relationship between tracer uptake and the secretin-induced volume of duodenal secretions as well as its amylase and HCO_3_^−^ content [10, 11].

Okazumi et al. [12] were the first to report a relationship between methionine uptake and the insulinogenic index, which is the relationship between fasting insulin and glucose and closely related to insulin resistance (IR). Recently, Otsuki et al. measured [^11^C]MET uptake in type I diabetics, showing a lower tracer uptake as compared to healthy controls. Moreover, in pancreas transplantation, a relationship was found between tracer uptake in the donor pancreas and the duration of insulin independence after transplantation. The authors also suggested this nuclear medical approach to evaluate prospective pancreas donors [5–7].

Neoplasms of the pancreas head are treated surgically by pancreaticoduodenectomy (PD) which comprises the resection of the pancreatic head, the duodenum and — in the Whipple procedure — distal gastrectomy. The pancreatic remnant, bile duct and stomach are then reconnected using a jejunal loop [13, 14]. Diabetes and impaired glycemic control is an issue in cancer of the pancreas, where new onset diabetes mellitus is observed [15–17], that frequently recedes after PD, the cause of which is not known [18]. The velocity of gastric emptying has been shown to instigate glycemic control in subjects having undergone PD [19]. Gastric emptying speed can be estimated by assessing the pace of paracetamol absorption, because paracetamol is not absorbed in the stomach. Its early postprandial plasma concentrations (AUC_30_) therefore represent the speed of gastric emptying [20, 21] a finding that has been validated by scintigraphy [22].

[^11^C]MET uptake seems to be a central indicator of beta cell function. Therefore, we looked for factors associated with the postprandial uptake in PD. We focused on possible relationships between gastric emptying, pre- and postprandial insulin concentrations, and [^11^C]MET uptake.

## Materials and methods

### Subjects

Nineteen tumor-free subjects after PD were chosen from the Medical University of Vienna’s surgical out-patient clinic after screening 81 patients for eligibility. Ten patients who had undergone a Whipple procedure and nine after pylorus-preserving PD (PPPD) were included. The underlying diseases had been neoplasms of the pancreas in 17 and chronic pancreatitis in two subjects. Cases with type I diabetes, renal failure, or cardiovascular diseases (>NYHA I) were not included. Patient characteristics and those of 10 healthy volunteers are listed in Table 1.

**Table 1:** Characteristics of patients after pancreaticoduodenectomy (PD) and healthy controls (median and 25/75 quantile)

	PD	Control	*p* value
Age (years)	64 (56 – 67)	24 (23.8 – 26)	<0.0001
Gender (M:F ratio)	1.11	2.33	n.s.
BMI (kg/m^2^)	22.8 (21.2 – 26.8)	23.7 (21.2 – 26.8)	n.s.
Time since operation (months)	18 (8 – 62)	-	-

### PET-CT imaging

[^11^C]MET was prepared on-site for a maximum of three consecutive patients [23]. When the subjects ingested the test meal, they simultaneously received an intravenous (i.v.) injection of 800 MBq [^11^C]MET and underwent the first of three PET-CTs after 15 minutes of accumulation. The process required three to five minutes per image. To reduce radiation exposure, only one primary CT scan was obtained to be fused with the subsequent PET scans. To obtain the best possible imaging quality, subjects remained in a supine position, moving as little as possible, until the last scan was recorded. At 15, 30, and 60 minutes after tracer application, PET scans were recorded.

The imaging was performed using an integrated PET/CT camera (Biograph 64 with True Point, Siemens, Erlangen, Germany) equipped with a full-ring dedicated PET scanner and a 64-slice CT scanner. CT scans were acquired with a 0.6-mm slice thickness and a rotational speed of 0.5 s/rotation, using a tube voltage of 120 kV and a nominal current of 230 mA and pitch of 0.8. CT images were reconstructed with the smoothing “standard” and the edge-enhancing “bone” algorithms.

The PET scanner was equipped with a 21.6-cm axial field of view. Imaging time was three minutes per bed position. Patients and controls were scanned over the abdomen region. Images were reconstructed in a 168 × 168 matrix with a 5-mm pixel size using a two-dimensional ordered subset, reconstruction true X, four iterations, and a 21 subsets all-pass filter. Attenuation correction was performed using the previously obtained CT scan reconstructed with an extended field of view. Images were processed with a smoothing filter assuming a full-width at half maximum (FWHM) of 5 mm (with a post-filter assuming a FWHM of 5 mm). After processing of stand-alone CT and PET images, a fusion data set with a transaxial slice thickness of 5 mm was assembled.

### Calculations

The area under the curve (AUC) was calculated using the trapezoid rule to retrieve integrated values of the measured parameters. Therefore, AUC over 30 minutes (AUC_30_) was calculated as plasma concentrations/activity at 0–30 minutes (C_0_-C_30_) and AUC over 60 minutes (AUC_60_) of concentration/activity at 0–60 minutes (C_0_-C_60_) after test meal ingestion: AUC_30_ = ((C_0_ + C_10_)*10/2) + ((C_10_ + C_20_)*10/2) + ((C_20_ + C_30_)*10 /2) and AUC_60_ = AUC_30_ + ((C_30_ + C_60_)*30/2). In the case of [^11^C]MET uptake: AUC_60_ = (C_15_ + C_30_)*15/2 + ((C30 + C60)*30/2). Delta represents the incremental growth over time and is the difference between concentrations of a measured parameter at two different time points.

### Imaging analysis

Maximum tissue standardized uptake values (SUVmax) were obtained manually, based on regions of interest over the pancreas and over the liver. The [^11^C]MET pancreas to liver ratio (PLR) was calculated. Regions of interest were the body and tail of the gland. Tracer uptake increments (delta-SUVmax) were calculated between 15–30 and 15–60 minutes. AUC_60_ of SUVmax was calculated as a measure of total uptake.

### Modeling analysis

The homeostasis model assessment (HOMA) was applied to calculate beta cell function and insulin resistance. The formula by Matthews et al. [24] is based on the physiological glucose production of the liver and the resulting insulin output of the beta cells of the pancreas, regulating blood sugar levels in a fasting state. Therefore, fasting plasma insulin (FPI, mU/l) and fasting plasma glucose (FPG, mmol/l) were used to calculate beta cell function (%B) and insulin resistance (IR) with the following formulas: HOMA1 - IR = (FPI x FPG)/22.5; and HOMA1 - %B = (20 x FPI)/(FPG - 3.5).

### Blood tests

After an overnight fast, an indwelling catheter was inserted into the cubital vein and blood was sampled before the ingestion of a liquid test meal (described below), as well as 10, 20, 30, 60, 90, 120, 150, and 180 minutes thereafter for the measurement of insulin, glucose, and paracetamol concentrations. Blood samples were obtained alongside the [^11^C]MET PET-CT scans.

Plasma glucose was measured by the glucose oxidase method, and insulin concentrations as described previously [25]. For the measurement of paracetamol, blood was collected in heparin-coated collection tubes. Acetaminophen was extracted with ethyl acetate and derivatized with N-methyl-N-(trimethylsilyl)trifluoroacetamide (MSTFA, Macherey & Nagel, Düren, Germany). D4-Acetaminophen (LGC Standards, Luckenwalde, Germany) was used as the internal standard. The calibration range was from 1 to 50 microgram/mL; the acetaminophen standard was obtained from Lipomed (Arlesheim, Switzerland). Analyses were performed by gas chromatography–mass spectrometry (Agilent) in the EI scan mode.

### Mixed test meal

All subjects ingested a liquid test meal after the fasting blood samples had been obtained. The meal was made up of 200 mL Fresubin Protein Energy© (Fresenius Kabi, Graz, Austria) and 50 mL cream (36 % fat). This liquid meal of 250 mL consisted of 26.5 g carbohydrates, 21.1 g proteins, 31.4 g fat, and a total of 472 kcal [26]. The drink was ingested within 1 minute. One g of paracetamol was admixed to the meal. Gastric emptying was estimated by calculating AUC_30_ from serial measurements of plasma paracetamol concentrations [20–22].

### Statistics

Variables are presented in median, 25 %, and 75 % quantiles. Using JMP version 9, comparisons between parameters were carried out using the nonparametric Wilcoxon test and the one-sided Fisher’s exact test. Univariate and multivariate linear regression was applied where appropriate. A *p* value < 0.05 was considered significant.

## Results

**Fig. 1:**
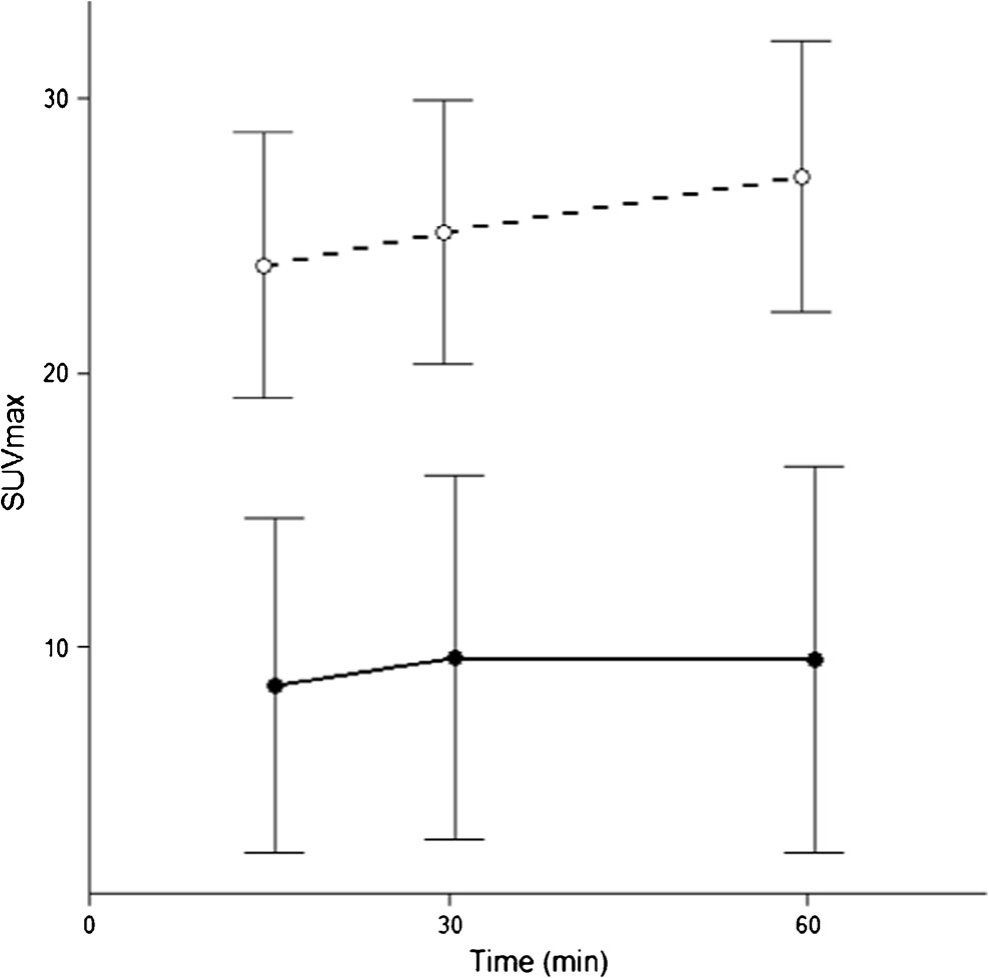
Maximal standardized uptake values (SUVmax) 15, 30, and 60 minutes after [*S-methyl*-^11^C]-L-methionine application in PD subjects (•, n = 19) and controls (°, n = 10; means and SD)

[^11^C]MET uptake was significantly higher in controls compared to patients, at any of the three time points of measurement (Table 2). Within the groups, the uptake did not differ between the time points (Fig. 1). In controls, one measurement of [^11^C]MET was not possible due to technical problems. In this subject, SUVmax AUC_60_ could not be calculated.

**Table 2:** Pancreas/liver-ratio (PLR) and maximum tissue standardized uptake values (SUVmax) of the pancreas after 15, 30, and 60 minutes, total integrated-SUVmax at 15–60 minutes (AUC_60_), and delta-SUVmax (15–30 and 15–60 minutes) in patients after PD and in controls (median and 25/75 quantile)

	PD	Control	*p* value
PLR			
15 30 60	0.61 (0.29 – 1.28) 0.68 (0.24 – 1.26) 0.61 (0.27 – 1.02)	2.27 (2.01 – 2.76) 2.51 (2.04 – 2.84) 2.35 (1.97 – 2.95)	<0.0001 <0.0001 <0.0001
SUVmax			
15 30 60	8.55 (2.7 – 12.5) 10.05 (2.9 – 13.9) 8.65 (3.1 – 15.3)	25.38 (19.33 – 27.49) 24.95 (22.76 – 28.78) 28.69 (23.42 – 30.53)	<0.0001 <0.0001 <0.0001
Delta-SUVmax			
30 60	0.70 (0.00 – 1.83) 0.91 (0.20 – 1.85)	1.77 (0.95 – 2.80) 3.29 (1.86 – 4.62)	n.s. 0.0073
SUVmax-AUC			
60	432.75 (144.38 – 626.25	1159.88 (1001.74 – 1324.80)	<0.0001

**Fig. 2:**
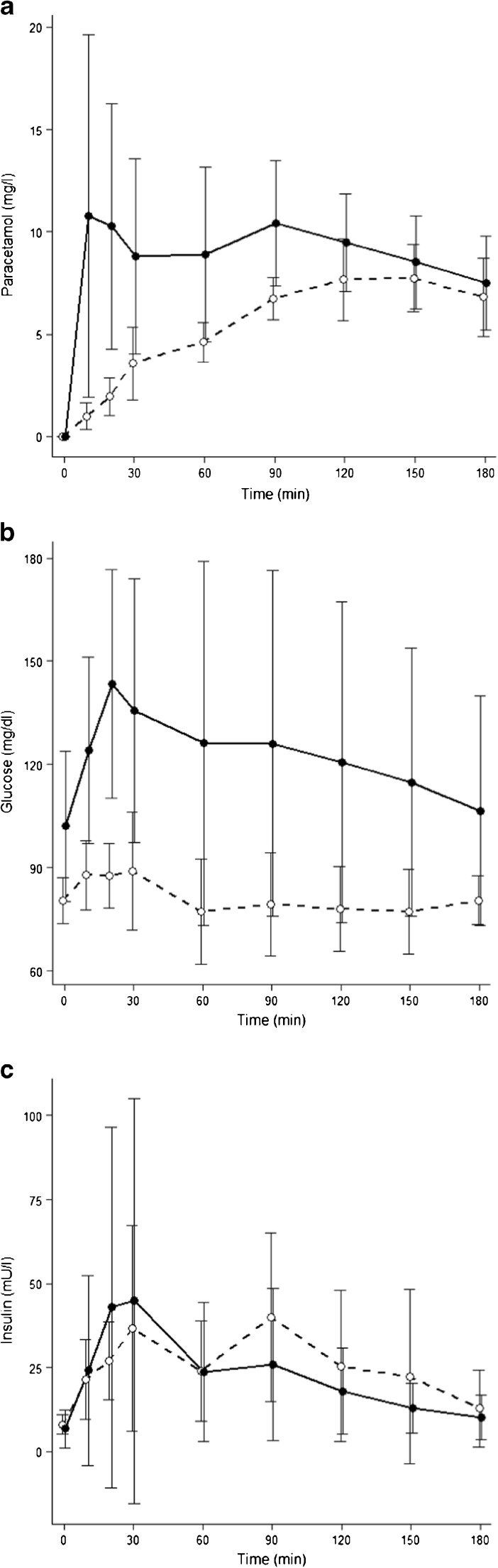
Time course of paracetamol (a, n = 18), glucose (b, n = 18), and insulin (c, n = 17) concentrations after the test meal in patients after PD (•) and in controls (n = 10,°; means and SD)

Paracetamol peak concentrations and AUC_30_ were significantly higher after PD (Table 3, Fig. 2a). Glucose baseline, peak concentrations, and AUC_60_ were higher in patients compared to controls, whereas insulin concentrations did not differ (Table 3, Fig. 2b, c). HOMA-derived beta cell function (%B) was smaller in PD subjects compared to controls whereas IR did not differ (Table 3). Due to problems in continuous blood aspiration and one corrupted insulin measurement, paracetamol measurements were analyzable in 18 patients, insulin concentrations in 17 patients, and glucose measurements in 18 patients. Single insulin parameters of one control were missing, in which case no insulin AUC_60_ could be calculated.

**Table 3:** Glucose, insulin, and paracetamol concentrations. Beta cell function (%B) and insulin resistance (IR; median and 25/75 quantile)

	PD	Control	*p *value
Paracetamol			
Peak (mg/l) Time to peak (min) AUC_30_(mg/l x min)	11.9 (10 – 25.4) 90 (10 – 127.5) 217.7 (128.9 – 367.8)	7.7 (6.8 – 11.4) 135 (112.5 - 150) 44 (37.8 – 57.8)	0.0017 0.0229 <0.0001
Insulin			
Baseline (mU/l) Peak (mU/l) AUC_60_(mU/l x min)	5.28 (2.9 – 8.82) 33.2 (22.1 – 74.45) 1067.7 (678.2 – 2550.58)	8.67 (5.5 – 10.7) 37 (28.7 – 84.15) 1283.75 (914.35 – 2267.58)	n.s. n.s. n.s.
Glucose			
Baseline (mg/dl) Peak (mg/dl) AUC_60_(mg/dl x min)	96.5 (85.3 – 110.8) 157.5 (132.3 – 171) 8005 (6025 – 8475)	82 (74.25 – 85) 97 (92 – 107.75) 4815 (4685 – 5570)	0.0014 0.0001 0.0004
HOMA			
%B IR	59.2 (25.77 – 109.22) 1.05 (0.81 – 2.37)	180 (102.76 – 243.96) 1.8 (1.06 – 2.06)	0.0020 n.s.

**Fig. 3:**
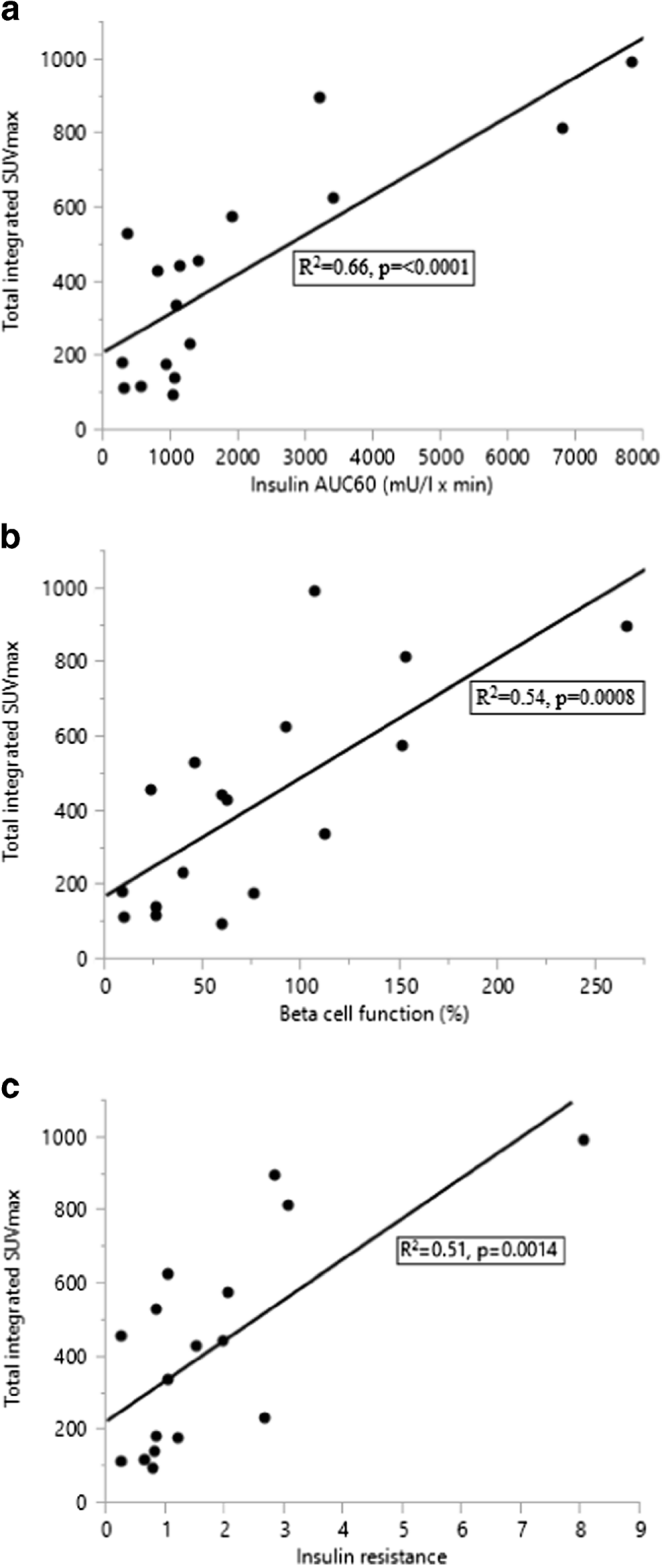
Relationship between total integrated SUVmax (AUC_60_) and insulin AUC_60_ (a), beta cell function (b), and IR (c) in patients after PD (•, n = 17)

**Fig. 4:**
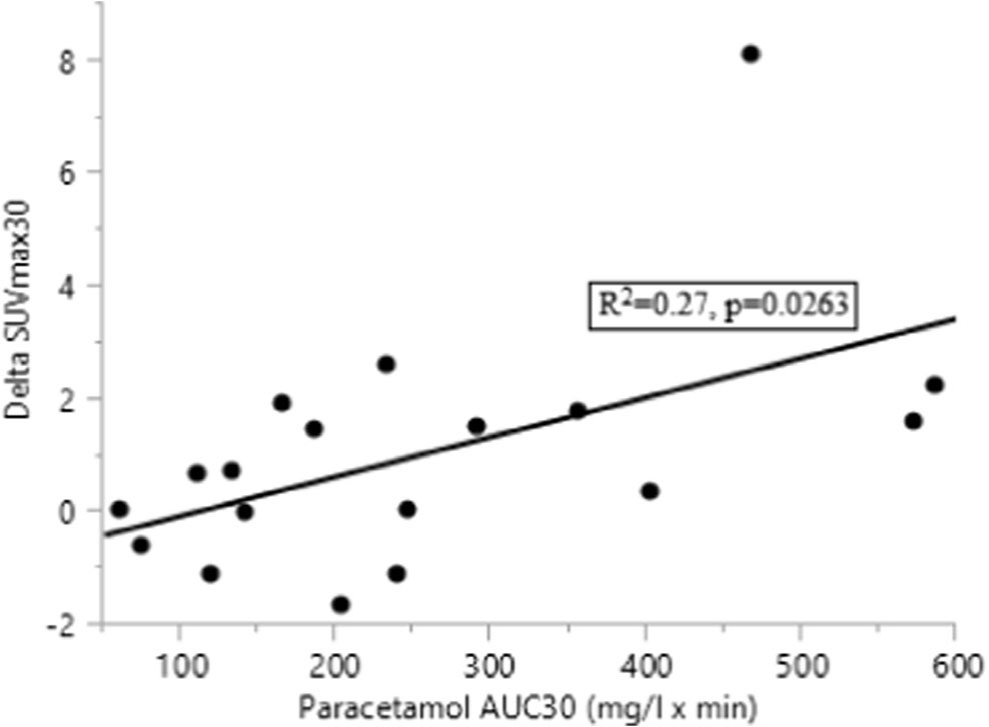
Gastric emptying (integrated paracetamol [AUC_30_]) and incremental SUV between 15 and 30 minutes post-meal in patients after PD (•, n = 18)

In patients, putative factors associated with total integrated SUVmax, such as BMI, age, insulin AUC_60_, %B, IR, and paracetamol AUC_30_ were evaluated by single linear regression and tested for significance. Insulin AUC_60_(Fig. 3a), beta cell function (Fig. 3b), and IR (Fig. 3c) correlated with SUV. The increment of SUVmax between 15 and 30 minutes (delta-SUVmax30) showed a weak correlation with gastric emptying (Fig. 4).

**Fig. 5:**
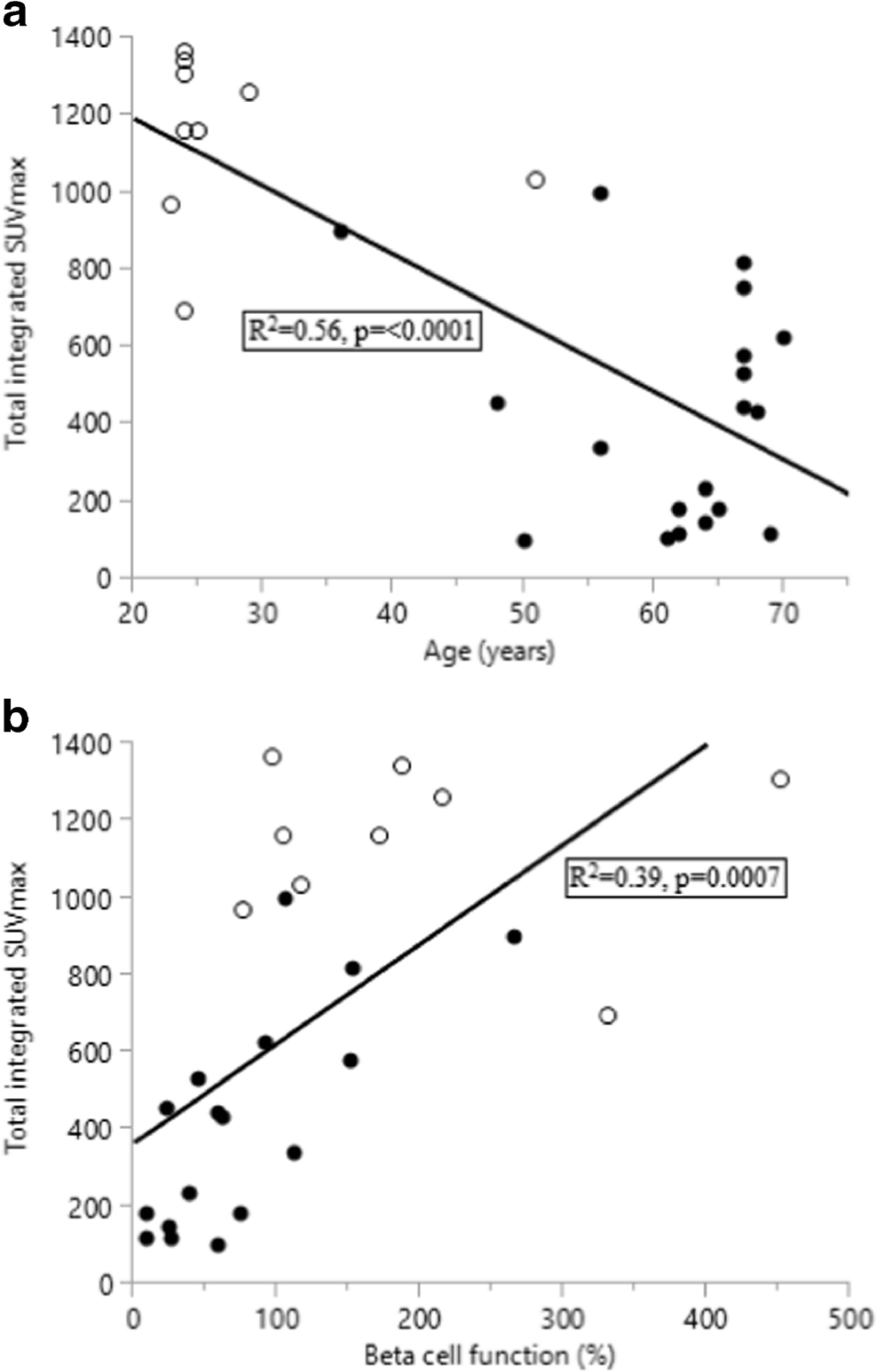
Relationship between total integrated SUVmax (AUC_60_), age (a, n = 19) and beta cell function (b, n = 17) in patients after PD (•) and in controls (°, n = 9)

Including controls in the analyses, age (Fig. 5a) correlated inversely and %B (Fig. 5b) correlated with total integrated SUVmax; other parameters were not significant.

In multivariate regression, integrated insulin (AUC_60_) and beta cell function emerged as independent predictors of ^11^C-methionine uptake, i.e., total integrated SUVmax (R^2^ = 0.78, *p* value = <0.0001, Table 4) in patients after PD. Including controls in multiple regression analysis, age and insulin AUC_60_ showed to be independent predictors of [^11^C]MET uptake (R^2^ = 0.69, *p* value = <0.0001, Table 4), i.e. age replaced %B when controls were included.

**Table 4:** Multiple linear regression on the dependent variable ‘total integrated SUVmax’ in patients after PD (n = 17; R^2^ = 0.78, *p* value = <0.0001) and PD patients (n = 17) plus controls (n = 8; R^2^ = 0.69, *p* value = <0.0001)

PD patients			
Variable	Estimate	Standard error	*p* value
Intercept	132.95	55.25	0.0305
%B	1.81	0.64	0.0137
Insulin AUC_60_	0.08	0.02	0.0013
No additional significance: paracetamol AUC_30_, BMI, age			
PD patients and controls			
Intercept	1396.07	150.57	<0.0001
Age	−17.91	2.73	<0.0001
Insulin AUC_60_	0.09	0.03	0.0036
No additional significance: paracetamol AUC_30_, BMI, %B			

## Discussion

PD subjects, both with or without pylorus preservation, had higher paracetamol concentrations (AUC_30_) compared to controls, suggesting more rapid gastric emptying in the PD patients regardless of pylorus preservation (PPPD). The higher fasting glucose in the PD patients probably reflects the lower steady-state beta cell function. The rapid postprandial rise of plasma glucose can be explained by the more rapid gastric emptying, a relationship that is also observed after operations such as total or partial gastrectomy [27, 28]. The composition of the test meal produced lower glucose concentrations compared to an oral glucose tolerance test used earlier [19]. The same test meal, however, in PD patients, at shorter intervals since operation than ours, showed a considerably lower increase of paracetamol, suggesting that the speed of gastric emptying recovers with time after the operation [26].

Compared to controls, the patients exhibited an impaired glycemic control: their baseline glucose concentrations were higher and baseline beta cell function was lower. The integrated glucose concentrations during the first hour (AUC_60_), when the [^11^C]MET uptake was measured, were twice the value of those in controls. Since our test meal contained no glucose and only 26 g carbohydrates compared with the 75 g glucose used in the oral glucose test by Harmuth et al. [19], we consider this difference to be a sign of impaired glycemic control rather than the consequence of rapid gastric emptying. The later peak of insulin concentrations in controls may also be explained by the slower gastric emptying. The failure of the PD patients’ early postprandial insulin to exceed that of controls is conceivably one of the sequelae of inadequate insulin production capability, a possible sequel of resection and advanced age.

The lower SUVmax in patients and the correlation between [^11^C]MET uptake and early insulin concentrations suggest that the tracer uptake represents insulin production. Moreover, the preprandial baseline beta cell function also correlates with SUVmax. Differences in age might contribute to the difference of tracer uptake and insulin production between controls and PD subjects [29].

In the PD patients, the increase in SUVmax between 15 and 30 minutes correlated with paracetamol AUC_30_. It is well known that rapid gastric emptying enhances the release of incretins, such as GLP-1, which, in combination with glucose, is a potent stimulus of insulin release [30]. This likely explains the relationship between tracer uptake and gastric emptying. The association of methionine uptake and insulin production is further underlined by the results of multiple regression analysis, where basal beta cell function and first-hour integrated insulin concentrations were both independently correlated with SUVmax AUC_60_.

In earlier reports, the uptake of methionine has been discussed in the context of exocrine pancreatic function [9, 11]. Syrota and Takasu et al. evaluated [^11^C]MET uptake of the pancreas in patients suffering from chronic pancreatitis and alcohol disease, showing a relationship between tracer accumulation and enzyme output of the pancreas as well as tracer incorporation into digestive enzymes. Chronic pancreatitis is a destructive disease that involves the exo- and endocrine pancreas; therefore, diabetes is a common complication [31]. While the authors assume [^11^C]MET PET as an appropriate tool for examining the exocrine gland, diabetes and impaired endocrine function is hardly addressed in these publications. However, beta cell function is probably also represented by methionine accumulation in the pancreas. In patients after PD, the exocrine pancreatic function is significantly impaired [32]. In our population, correlations between beta cell function and total methionine uptake were significant. We found a close association between uptake and basal as well as postprandial beta cell function, but we cannot determine the relative contribution of the exocrine function to the measured uptake. As already mentioned above, Otsuki et al. identified a reduced uptake of methionine in pancreata of patients with type I diabetes. They also showed that after pancreas transplantation, the tracer uptake in the donor pancreas predicted the long-term efficacy of the transplant to maintain glycemic control [2, 5–7], stressing the significance of beta cell function for the uptake.

Not differentiating between exo- and endocrine pancreatic function, Kono et al. [4] measured methionine uptake before and after operation in patients undergoing distal pancreatectomy and in PD cases. The fasting methionine uptake in the remaining pancreas was lower in the PD group, the cause of which is unknown. The lower methionine uptake after PD, compared to left-sided pancreas resection, might be explained by the lower IR as a result of malabsorption and ensuing malnutrition after PD [33, 34]. Conceivably, the lower uptake in PD patients could also be a sign of impaired stimulation of the exocrine pancreas after duodenal resection and reduced release of hormones that stimulate the exocrine gland, such as cholecystokinin [32].

Unfortunately, our results do not allow us to determine the relative impact of exocrine and endocrine function, and further studies with simultaneous measurement of both functions seem to be desirable to clarify the correlation between [^11^C]MET uptake and insulin production. One major limitation of this study was the younger control group of healthy subjects, which prevented us to assess the impact of age on the measurements.

This study, the first to look at [^11^C]MET uptake in combination with plasma insulin concentrations during a test meal, illustrates the relationship between tracer uptake and insulin production. The findings suggest that this imaging procedure is an appropriate tool to further study beta cell function.
